# SOCIAL RESOURCES AND STRATIFIED EXPERIENCES OF ICT USE AND LONELINESS DURING THE COVID-19 PANDEMIC

**DOI:** 10.1093/geroni/igac059.125

**Published:** 2022-12-20

**Authors:** Sungjae Hong, Shannon Mejía

**Affiliations:** University of Illinois at Urbana-Champaign, Champaign, Illinois, United States; University of Illinois at Urbana-Champaign, Champaign, Illinois, United States

## Abstract

Limits to in-person social interaction increased the risk of loneliness during the Covid-19 pandemic. This study examines how gender and previous information communication technologies (ICT) experience could stratify how ICT-mediated social interactions related to loneliness during the COVID-19 pandemic. We employed National Health & Aging Trends Study data collected in 2018 and 2020-2021 (N=2,962; 27% age 85+; 57% women; 77% white) to examine how the email and video call use before and during the COVID-19 pandemic was associated with loneliness and how the degree of impact differed by gender and previous ICT experience. In total, 35% reported feeling lonely some days or more frequently during the COVID-19 pandemic. Preliminary analysis showed older adults using video calls during the COVID-19 pandemic were 1.24 times more likely to report loneliness. This association was stronger among men than women and those who did not use ICT preceding the pandemic than those who used.

